# Dreaming Ubuntu: Jungian Studies, Forgiveness, and Jung’s Recalcitrant Fourth

**DOI:** 10.1111/1468-5922.13079

**Published:** 2025-02-10

**Authors:** Barbara Cerminara

**Affiliations:** ^1^ Norwich UK

**Keywords:** cultural superego, forgiveness, reconciliation, resentment, South Africa, Truth and Reconciliation Commission (TRC), Tutu, Ubuntu, Ubuntu, Commission Vérité et Réconciliation, Tutu, ressentiment, réconciliation, pardon, Afrique du Sud, surmoi culturel, Ubuntu, Wahrheits‐ und Versöhnungskommission, Tutu, Groll, Versöhnung, Vergebung, Südafrika, kulturelles Über‐Ich, Ubuntu, Commissione per la Verità e Riconciliazione, Tutu, rancore, riconciliazione, perdono, Sudafrica, super‐Io culturale, убунту, Комиссия по установлению истины и примирению, Туту, возмущение, примирение, прощение, Южная Африка, культурное суперэго, Ubuntu, Comisión por la Verdad y la Reconciliación, Tutu, resentimiento, reconciliación, perdón, Sudáfrica, superyó cultural, 乌班图, 真相和解委员会, 图图, 怨恨, 和解, 宽恕, 南非, 文化超我

## Abstract

Jung’s recalcitrant fourth comes in from a place of opposition, demanding that what has been neglected be considered. It is in the spirit of the fourth that the author examines the use of Ubuntu in Jungian literature, cautioning against a decontextualized appropriation of the notion that overlooks its diverse interpretations and usages, particularly in terms of its role in shaping the new South Africa. The author notes a tendency among Jungian scholars to view Ubuntu as the *exotic Other*, inadvertently perpetuating the same colonial mindset that Jung held towards non‐European cultures. Moreover, she suggests that the Jungian viewpoint on Ubuntu has been heavily influenced by Desmond Tutu’s Christianized version of the notion, and that this assimilation holds significant implications. As chair of the Truth and Reconciliation Commission, the archbishop employed the idea of Ubuntu in the service of nation building. Ubuntu was part of a political strategy designed to reconcile the South African people, whilst also silencing critics of a process that failed to fully address structural injustices. Now assimilated into Jungian literature, *this* version of Ubuntu took on aspects of a punitive cultural superego, thereby undermining its original appeal as an autochthonous form of humanism invoked to liberate the oppressed.

## Abstract (fr)

Selon Jung son Quatrième Récalcitrant surgit à partir d’un point d’opposition, tel qu’il exige à ce qu’il soit pris en compte. C’est dans l’esprit de ce quatrième que l’auteur interroge l’utilisation de l’Ubuntu dans la littérature jungienne, mettant en garde une appropriation décontextualisée de cette notion qui négligerait ses diverses interprétations et mises à l’œuvre, en particulier concernant son rôle dans le façonnement de la nouvelle Afrique du Sud. L’auteur note une tendance chez les chercheurs jungiens à considérer l’Ubuntu comme l’Autre Exotique, perpétuant par inadvertance la même mentalité coloniale, que celle manifestée par Jung à l’égard des cultures non‐européennes. En outre, elle suggère que le point de vue jungien sur l’Ubuntu a été fortement influencé par une version christianisée de la notion par Desmond Tutu, et que cette assimilation relève d’implications signifiantes. En tant que président de la Commission Vérité et Réconciliation, l’archevêque a mis l’idée d’Ubuntu au service de la construction de la nation. L’Ubuntu a fait partie d’une stratégie politique destinée à réconcilier le peuple sud‐africain, tout en réduisant au silence les détracteurs d’un processus qui ne s’est pas attaqué pleinement aux injustices structurelles. Désormais assimilé à la littérature jungienne, *cet* Ubuntu a pris des traits d’un surmoi culturel punitif, sapant ainsi son attrait initial en tant que forme autochtone d’humanisme invoquée à libérer les opprimés.

## Abstract (de)

Jungs widerspenstiger vierter Ansatz kommt aus einer Position der Opposition und fordert, daß das Vernachlässigte berücksichtigt werde. Im Geiste des vierten Ansatzes untersucht die Autorin die Verwendung von Ubuntu in der Jungianischen Literatur und warnt vor einer dekontextualisierten Aneignung des Begriffs, die seine vielfältigen Interpretationen und Verwendungen übersieht, insbesondere im Hinblick auf seine Rolle bei der Gestaltung des neuen Südafrika. Die Autorin stellt fest, daß Jungianer dazu neigen, Ubuntu als das *exotische Andere* zu betrachten, und damit unbeabsichtigt dieselbe koloniale Denkweise fortführen, die Jung gegenüber nichteuropäischen Kulturen hatte. Darüber hinaus weist sie darauf hin, daß die Jungianische Sichtweise auf Ubuntu stark von Desmond Tutus christianisierter Version des Begriffs beeinflußt wurde und daß diese Assimilation erhebliche Auswirkungen hat. Als Vorsitzender der Wahrheits‐ und Versöhnungskommission setzte der Erzbischof die Idee von Ubuntu in den Dienst des Nation Building. Ubuntu war Teil einer politischen Strategie, die darauf abzielte, das südafrikanische Volk zu versöhnen und gleichzeitig Kritiker eines Prozesses zum Schweigen zu bringen, dem es nicht gelang, strukturelle Ungerechtigkeiten vollständig beseitigen. *Dieses* Ubuntu wurde inzwischen in die Jungianische Literatur aufgenommen und nahm Aspekte eines strafenden kulturellen Über‐Ichs an, wodurch seine ursprüngliche Anziehungskraft als autochthone Form des Humanismus, die zur Befreiung der Unterdrückten aufruft, untergraben wurde.

## Abstract (it)

Il recalcitrante quarto di Jung emerge da una opposizione, chiedendosi cosa è stato trascurato. È nello spirito del quarto che l'Autrice esplora l'uso dell'Ubuntu nella letteratura junghiana, avendo cautela nel decontestualizzare la nozione per non lasciarsi sfuggire le sue diverse interpretazioni ed utilizzi, in particolare per quanto riguarda il suo ruolo nel definire il nuovo Sudafrica. L'Autrice nota una tendenza negli studiosi junghiani che vedono l'Ubuntu come *l'Altro esotico*, inavvertitamente perpetuando la stessa mentalità coloniale che Jung aveva verso le culture non europee. Inoltre, suggerisce che la prospettiva junghiana sull'Ubuntu sia stata pesantemente influenzata dalla versione cristianizzata che ne ha fatto Desmond Tutu, e che questo abbia avuto importanti implicazioni. Come Capo della Commissione per la Verità e la Riconciliazione, l'arcivescovo ha utilizzato l'idea dell'Ubuntu per la costruzione della nazione. L'Ubuntu ha fatto parte della strategia politica sviluppata per riconciliare la popolazione sudafricana, mentre ha anche permesso di silenziare le critiche ad un processo che ha, in realtà, fallito la piena denuncia delle ingiustizie strutturali. Assimilato nella letteratura junghiana, *questo* Ubuntu assume aspetti di un super‐Io culturale punitivo, così sminuendo il suo fascino originario come forma autoctona di umanesimo, invocato per liberare gli oppressi.

## Abstract (ru)

Четверичность Юнга строится на принципе оппозиции, требующей внимания к тому, чем ранее пренебрегали. Руководствуясь принципом четверичности, автор рассматривает использование понятия убунту в юнгианской литературе и предостерегает от его употребления вне контекста, без учета различных его интерпретаций и значений, особенно в плане его роли в формировании новой Южной Африки. Автор отмечает склонность юнгианских исследователей трактовать убунту как экзотическое *иное* и таким образом непреднамеренно закреплять колониальные установки, свойственные Юнгу по отношению к неевропейским культурам. Более того, автор предполагает, что на юнгианские представления об убунту существенно повлияла трактовка этого понятия в духе христианства Десмондом Туту. Будучи председателем Комиссии по установлению истины и примирению, он использовал идею убунту в целях формирования национальной идентичности. Убунту стало частью политической стратегии, направленной одновременно на примирение населения Южной Африки и на усмирение критиков неудачных попыток преодоления социальной несправедливости. В настоящее время понятие убунту ассимилировалось в юнгианской литературе, где оно приобрело черты карательного культурного суперэго и утратило свое первоначальное позитивное значение автохтонной формы гуманизма, призванной освободить угнетенных.

## Abstract (es)

El recalcitrante cuatro de Jung entra desde un lugar de oposición, exigiendo que se considere lo que ha sido negado. Es en el espíritu del cuatro que la autora examina el uso de Ubuntu en la literatura Junguiana, advirtiendo contra una apropiación descontextualizada de la noción que pasa por alto sus diversas interpretaciones y usos, particularmente en términos de su papel en la configuración de la nueva Sudáfrica. La autora observa una tendencia entre los estudiosos de Jung a considerar Ubuntu como el *Otro exótico*, perpetuando inadvertidamente la misma mentalidad colonial que Jung tenía hacia las culturas no europeas. Además, sugiere que el punto de vista Junguiano sobre Ubuntu ha sido fuertemente influenciado por la versión cristianizada de la noción de Desmond Tutu, y que esta asimilación tiene implicancias significativas. Como presidente de la Comisión por la Verdad y la Reconciliación, el arzobispo empleó la idea de Ubuntu al servicio de la construcción nacional. Ubuntu formaba parte de una estrategia política concebida para reconciliar al pueblo sudafricano, al tiempo que silenciaba las críticas de un proceso que fallaba en abordar las injusticias estructurales. Asimilado ahora a la literatura Junguiana, *este* Ubuntu adoptó aspectos de un superyó cultural punitivo, socavando así su atractivo original como forma autóctona de humanismo invocada para liberar a los oprimidos.

## Abstract (zh)

做梦的乌班图:荣格学研究、宽恕和荣格的顽固第四

荣格的“顽固的第四”从相反的角度出发, 要求我们考虑被忽视的东西。作者正是本着“第四”的精神, 研究了荣格文献中对“乌班图”的使用, 告诫人们不要脱离实际地使用这一概念, 而忽略了它的多种解释和用法, 尤其是它在塑造新南非方面的作用。作者指出, 荣格学者倾向于将“乌班图”视为异域他者, 无意中延续了荣格对非欧洲文化持有的殖民思维。此外, 她还指出, 荣格关于“乌班图”的观点深受德斯蒙德﹣图图基督教化版本的“乌班图”概念的影响, 这种同化具有重大意义。作为真相和解委员会的主席, 这位大主教将“乌班图”理念用于国家建设。“乌班图”是一项政治战略的一部分, 旨在实现南非人民的和解, 同时压制对于结构性不公正问题无法彻底解决的问题的批评。现在, “乌班图”已被荣格文献吸收, 这一“乌班图”具有了惩罚性文化超我的某些面向, 从而削弱了其作为一种被用来解放被压迫者的本土人文主义形式的原有吸引力。

## Introduction

As he enlists Gilbert Simondon, a philosopher, to expand on Jung’s concept of individuation, Mark Saban writes:

I am a Jungian analyst and not a philosopher. I am very aware that this means I am in danger of doing what Jungians (and Jung himself) have historically been very good at doing: colonizing alien disciplines, appropriating (and thereby distorting) the fruits of those disciplines, and forcing the whole into service for the greater good of Jungian psychology. The pages of Jungian journals are littered with half‐digested, half‐thought neuroscience, quantum physics, post‐modern philosophy—in much the same way they used to be strewn with barely understood ideas from anthropology, ethology, and theology. (2019, p. 91)

This paper pays heed to Saban’s warning, cautioning against a decontextualized appropriation of the notion of Ubuntu in Jungian literature that overlooks its diverse interpretations and usages, particularly with regards to the role that this philosophical idea and living discipline played in the shaping of the new South Africa. I highlight a certain tendency among Jungian scholars to approach Ubuntu as the *exotic Other*, thus inadvertently perpetuating the same colonial attitude that Jung had towards non‐European cultures. Further, I suggest that Jungian perspectives on Ubuntu have been greatly influenced by Desmond Tutu’s Christianized version of the notion, and that this assimilation carries significant implications. This, especially since, as chair of the South African Truth and Reconciliation Commission (TRC), the Anglican archbishop employed the idea of Ubuntu in the service of nation building. Ubuntu, in other words, was incorporated into a political strategy designed to bring together and reconcile the South African people, while also silencing those who were critical of a reconciliation that failed to address the structural injustices the anti‐apartheid struggle aimed to redress. Tutu’s Christianized and globalized variety lost its local identity (the ubuntu with a lower case “u”), but it retained enough symbolic power to resonate with most of the black population who felt compelled to forgive and reconcile even in the absence of justice, and redistribution of land and wealth.

Jung (1948) ascribed the “quality of *realness*” to a fourth element which he contended was lacking in the trinitarian symbolism (Giegerich, 2020, p. 92, italics mine). This fourth element he called the *recalcitrant fourth*.

Jung’s fourth is often avoided because of its difficult nature. However, Jung suggests that the fourth should be given thorough consideration as it offers valuable psychological insights. Described as “an awkward customer” (para. 244), Jung maintains that the fourth is “particularly instructive” (para. 281) as it challenges consciousness to acknowledge those aspects of existence that do not align with our conscious aspirations and intentions. Coming in from a place of opposition and refusing to “come along with the others” (para. 245), the fourth demands that what has been left out be considered if not integrated. It is in the spirit of the fourth that I examine the use of Ubuntu in Jungian literature. 1

I begin with a brief overview of Ubuntu and its significance in the shaping of post‐apartheid South Africa, and then move on to discuss its use in Jungian scholarship. I conclude with some reflections on the idea of untimely forgiveness in relation to the works of the TRC.

My argument is that by focusing on forgiveness through the lens of Ubuntu, the TRC and its chair inadvertently transformed this ancestral southern African tradition into a tool of oppression that ultimately upheld the existing power structure rather than subverting it. Now assimilated into Jungian literature, *this* version of Ubuntu took on aspects of a punitive cultural superego, thereby undermining its original appeal as an indigenous form of humanism invoked to liberate the oppressed.

## Ubuntu: From Inclusivity to Exclusion

In years to come South African society may yet have to pay the price for the massive and manipulative repression of resentment and anger caused by the historically questionable use of ubuntu in the context of the TRC. (Binsbergen, 2001)

A system of values predominant among the Bantu‐speaking people living below the Sahara, Ubuntu (usually translated as *humanity* or *humanness*) “articulates a basic respect and compassion for others” (Louw, 2006, p. 161). The idea of Ubuntu is generally captured by the traditional African aphorism *a person is a person through persons*, a maxim which, explains South African philosopher Motsamai Molefe, points to “the social and relational nature of personal identity” (2020, p. 1). To quote Molefe:

Social relationships play an instrumental role in the pursuit or achievement of ubuntu. The moral logic in the discourse on ubuntu is that human beings can achieve personhood (ubuntu) only in and through social relationships. As such, to say someone has ubuntu is to make a moral judgment about the quality of their humanity which is typically understood in terms of perfection or moral virtue. (p. 1, see also Molefe, 2014)

As a type of social ethics, Ubuntu “not only describes human being as ‘being‐with‐others’, but also prescribes how we should relate to others: that is, what ‘being‐with‐others’ should be all about” (Louw, 2006, p. 161). Centring around the idea of group cohesion and consensus through dialogue, the practice of Ubuntu is assumed to “safeguard the rights and opinions of individuals and minorities” (p. 163). However, it has been noted that the idea of group loyalty may be enforced on those who fail to conform, a failure that can be met with harsh punishment. In these cases, Ubuntu is thought to legitimize a coercive praxis which favours sameness over difference. South African intellectual Themba Sono puts it this way:

The role of the group in African consciousness could be overwhelming, totalistic, even totalitarian. Group psychology […] pretends universality. This mentality, this psychology is stronger on belief than on reason; on sameness than on difference. Discursive rationality is overwhelmed by emotional identity, by the obsession to identify with and by the longing to conform to. To agree is more important than to disagree; conformity is cherished more than innovation. Tradition is venerated, continuity revered, change feared and difference shunned. Heresies are not tolerated in such communities. (cited in Louw, 2001, pp. 19–20; see also Louw 1998, 2006)

As a living discipline, thus, Ubuntu is not “infinitely accommodating, [and] not without boundaries” (Binsbergen, 2001, p. 430). Indeed, both African and non‐African scholars have highlighted the risks of Ubuntu becoming a seat of exclusion, a rejection according to which those whose life choices contrast with the communitarian spirit of Ubuntu, 2 or those who are not considered to be the legitimate heirs of a tradition “taken to be natural to the people of sub‐Saharan Africa” (Matolino & Kwindingwi, 2013, p. 203), become non‐persons, estranged from the human community. These are cases in which, Hanneke Stuit writes, the famous dictum “I am a person because of other persons” turns into a more sinister “I am a person because of other persons like me” (2010, p. 97).

Scholars have highlighted the “fundamental ambiguity that marks ubuntu” (Stuit, 2013, p. 13). Wrapped into cultural nationalistic discourses which are inherently conformist (Marx, 2002), as well as Afrocentrism, 3 and narratives of return (the yearning for the restoration of an imaginary pre‐colonial pristine existence characterized by order and harmony), 4 Ubuntu is considered by some to be an elitist project which reinforces the idea of a “we” as opposed to an alien “they”, a divide that inevitably invites exclusion (Marx, 2002).

Conceived in this way, this hallmark of inclusivity, tolerance, and solidarity towards the stranger is in danger of becoming the unlikely host of xenophobic leanings that mark as alien that which is thought to be in opposition with the communitarian ethics of an idealized version of the southern African village. In this case, tradition and culture are mobilized as ideological weapons that legitimize a climate of discrimination, with Ubuntu becoming the referent through which to determine who belongs and who does not (Stuit, 2010, 2013). And indeed, we learn from Thaddeus Metz, a prominent scholar of Ubuntu, that African ethics comprises the Bantu‐speaking peoples residing in sub‐Saharan Africa and yet it *excludes* “Islamic Arabs in North Africa and white Afrikaners in South Africa, *among others*” (2007, p. 321, italics mine). With these premises, writes Matolino, it is not surprising to learn that South Africa, “the home of ubuntu, and Johannesburg, a place where Metz argues all values of ubuntu can be animated, have earned a special place among notorious spots on the continent with an uncanny display of xenophobia” (2015, p. 219).

The exclusionary and conformist elements of Ubuntu may have seeped into the dominant narrative of reconciliation promoted by the key figures of post‐apartheid South Africa, including Tutu, who served as the chair of the TRC. It is possible that in the name of Ubuntu black South Africans felt obliged to let go of their resentment and extend forgiveness towards their former oppressor.

## Tutu’s Ubuntu

Underpinning the establishment of the South African Truth and Reconciliation Commission (TRC), the archbishop’s version of Ubuntu became instrumental to the building of the nation, a rainbow nation constructed “on the fantasy of harmony, equality and sameness” (van der Walt et al., 2003, p. 263) in the aftermath of apartheid (Wilson, 2001). “There is a need of understanding but not for vengeance, a need for reparation but not for retaliation, a need for *ubuntu* but not for victimization” (cited in Wilson, 2001, pp. 9–10, italics in original), recited the well‐known passage of the 1993 Interim Constitution of the Republic of South Africa (where the term first appeared). In post‐apartheid South Africa, Ubuntu thus became a sort of multicultural unifier and ideological marker creating the image of a compassionate African community that rejected the idea of retribution, favouring instead forgiveness and reconciliation.

Saturating the notion of Ubuntu, these African values were skilfully pitted against the Western morals of revenge and retributive justice by politicians and Tutu himself, closing down, regrettably, any conversation as to whether reconciliation could have been achieved through legal retributive processes. 5 As we shall see, Jungian scholars have followed in Tutu’s steps in their dismissal of retributive approaches to justice, with some authors confusing legal retribution with revenge. 6

A hybrid between African traditions and Christian customs of confession and forgiveness, the Ubuntu of the TRC left those who wished to see their perpetrators receive their just deserts, such as the family of activist Steve Biko (1946‐1977), feeling ignored and isolated. If the “TRC’s codified message was, ‘become a member of the New South Africa, or become incomprehensible in it’” (van der Walt et al., 2003, p. 259), then incomprehension was the price these families paid. The pressure to forgive and reconcile also created a split between the redeemed victim and the resentful victim, a rupture the consequences of which are still visible in present‐day South Africa (Cerminara, 2024; Meister, 2011; Rothfield, 2011). Indicative is the fact that the TRC has been accused of having facilitated a “sophisticated amnesia of the greater historical and structural violence of apartheid” (van der Walt et al., 2003, p. 251), thus contributing to a seamless transition from racial capitalism to a global capitalism that has left most of the black population poorer than it was during apartheid (Posel, 2014; Stone, 2004). Molefe puts it this way:

The TRC represents a machinery that engineered a political condition that imbued African people with an exceptional, accepting and forgiving humanity; this happened at the cost of sweeping the injured humanity of African people under the rug and protecting the material status quo. It is for this reason that, twenty years into democracy, there still exists a different spatial occupation for different races and economic inequalities between different racial groupings. (2014, p. 159)

It is crucial to note at this point that the TRC played a vital role in facilitating South Africa’s peaceful transition to democracy. Although a comprehensive analysis of the TRC’s initiatives aimed at reducing political violence and preventing a potential civil war goes beyond the scope of this discussion, 7 its noteworthy contributions merit acknowledgment. This is particularly important given recent scholarship challenging the dominant narrative of the South African “miracle” and accusing Nelson Mandela of a historic betrayal (Bundy 2019, p. 998; Bundy & Beinart, 2021). This contemporary critical perspective also challenges the TRC’s endorsement of a specific, Christianized, and globalized interpretation of Ubuntu. Critics argue that this interpretation may have inadvertently served as a tool of oppression and exploitation, thereby compromising the core principles of the anti‐apartheid movement. These are pressing matters that have received little attention in the Jungian discussion of Ubuntu, if at all.

## The Jungian Ubun Tutu

Just like Jung’s concept of transcendent function, the African notion of Ubuntu has gained traction among Jungian scholars because of its emphasis on interconnectedness and harmonious living rooted in forgiveness and reconciliation (Berg, 2004, 2012; Birgit Heuer, 2010a, 2011; Brooke, 2008, 2019; Donleavy & Shearer, 2008; Gottfried Heuer, 2010b; Shearer, 2008; Singer, 2016; Vaughan, 2019). In the maxim “I am because we are”, coined by Kenyan‐born Christian philosopher and writer John Mbiti, Jungians see an alternative to the Western conception of personhood captured by Descartes’ dictum “I think therefore I am”. 8 If the Western is individualist, then the African will affirm her communitarian identity in relation and opposition to the Western one (Molefe, 2014).

Jungian scholars have posited that Ubuntu could serve as a compensatory force to Western fragmentation and individualism. South African Jungian analyst Astrid Berg (2004, 2012), for instance, highly praises the shared sense of community and tolerance characteristic of Ubuntu humanism.

Ubuntu is also enlisted to counter Jung’s Eurocentric and discriminating perspective (Brooke, 2015, 2019), as well as Jung’s persistent distrust towards collectives which Jung thought would suffocate “the urge for individuation” (Brooke, 2008, p. 48). 9 Roger Brooke (2019), however, cautions that Jung’s injurious colonial attitude towards Africa, blackness and non‐whites in general, remains part of our inheritance as Jungians, a sort of “negative possession” if we were to use Jean Améry’s words (1980, p. 77), with which the Jungian community must wrestle. Brooke’s reminder echoes Renos Papadopoulos’ repeated warnings against a tendency to uncritically accept Jung’s idealized approach towards primitiveness. It should not be forgotten, writes Papadopoulos,

that Jung’s thinly disguised “noble savage” dogma about the “exotic other” constituted standard Jungian teaching until fairly recently; it was blindly adopted and widely applauded by most Jungians, blissfully unaware not only of its gross erroneousness but also of its dangerous colonial and racist implications. (2023, p. xxvi)

Arguably, the Jungian representation of Ubuntu holds remnants of this idealization of the *exotic Other*. For instance, Berg boldly claims that Ubuntu “starts from the personal and extends to the beyond to embrace the stranger and the whole of humanity in the way that Tutu has suggested” (2012, p. 99). This statement from Berg highlights a tendency not only to idealize Ubuntu, but also to universalize and decontextualize its significance.

The risk arising from certain descriptions of Ubuntu we trace in Jungian literature is that of reducing African reality to a monolithic narrative, a “single story” as Nigerian writer Chimamanda Ngozi Adichie calls it, 10 unable to contain the many stories of a diverse African society. As South African philosopher Bernard Matolino writes, “there is no known African essence” (2015, p. 220), meaning that the rich and multifaceted African world can’t be boxed into a single concept, not even one as appealing as Ubuntu.

However, Berg’s statement is significant as it demonstrates the impact that Tutu’s formulation of Ubuntu has had on the author’s perception of this particular form of African humanism. Indeed, as with other Jungian scholars, Berg’s interpretation of Ubuntu appears to be heavily influenced by the Christian rhetoric of the Anglican archbishop, and by the politics of the TRC. 11 In fact, what Berg and others seem to advocate for is a specific form of Christianized and globalized Ubuntu also known as “Christianized Ubun Tutu” (Praeg, 2014, p.76).

This variety of Ubuntu is indistinguishable from the forgiveness Tutu promoted as chair of the TRC, and indeed the two concepts – Ubuntu and forgiveness – are interchangeable when it comes to the work of the Commission. Telling, however, is the fact that in the pages of the National Unity and Reconciliation Act of 1995 which established the Commission, forgiveness was not mentioned, not even once. 12 Forgiveness, in other words, was Tutu’s project, as we can see from the title of his memoir, *No Future without Forgiveness*. The archbishop, as Jacques Derrida put it, “Christianized the language of an institution”, introducing “the vocabulary of repentance and forgiveness” in the political process of national reconciliation (2001, p. 42; see also Smith, 2008).

Joined together, forgiveness and reconciliation emerged as the hallmark of the South African TRC, with Ubuntu becoming a synonym for both. It must be noted, however, that while forgiveness is often linked with reconciliation, the former is not necessarily a component of the latter. People may be able to coexist without having to let go of their resentment. As professor of public politics David Crocker suggests, “[r]econciliation as nonlethal coexistence […] demands significantly less and is easier to realize than Tutu’s much ‘thicker’ ideal that requires friendliness and forgiveness” (2002, p. 528). 13

Crocker’s argument resonates with Jung’s emphasis on the importance of completeness rather than perfection. Jung articulates this idea by stating that “there is no […] psychic wholeness *without* imperfection. To round itself out, life calls not for perfection but for completeness” (1936, para. 208, italics mine). Efforts to reconcile divided societies should, in my view, prioritize pragmatic good‐enough reconciliation over idealistic models based on forgiveness (Cerminara, 2024; Samuels, 2010, 2013). 14

Tutu’s idealized version of Ubuntu centred on the loving side of human relating, overlooking aspects of the ubuntu with the lower case “u” that are believed to survive nowadays in rural southern Africa. These include methods of “benevolent coercion, indoctrination, [and] inculcation” aimed at transforming an individual into a person with ubuntu (Praeg, 2014, p. 65). With its praxis of communal obligations and distribution of labour, this down‐to‐earth version of ubuntu delimits the conditions of belonging to those who have been taught the values of ubuntu, ultimately setting itself at odds with the profoundly Christian idea of universal shared humanity advocated by Tutu and the TRC and reaffirmed in Jungian literature.

## Ubuntu as the Good News

Following Tutu closely and often quoting at length directly from his work, Jungian scholars appear to have conflated Ubuntu with the “good news” (Praeg, 2014, p. 66; Binsbergen, 2001), describing it, as we shall see, as a wholesome Other, the Other which holds the promise of *coniunctio* in a divided and battered world. The contention here is not unlike that of Professor Martin Prozesky, who declares: “African ethics can indeed be seen as a salvatory power in today’s heartlessly globalizing world, a potential moral saviour in a time of deep trouble” (cited in Praeg, 2014, p. 116). Yet, according to anthropologist Wim van Binsbergen, what Prozesky believes is Africa’s greatest gift to humanity is in effect:

… the gift of the academic and managerial codifiers who allowed themselves to be distantly and selectively inspired by village life: ignoring the ubiquitous conflicts and contradictions […] and *merely appropriating and representing the bright side*. (2001, pp. 447–448, italics mine)

Binsbergen’s contention may indeed reveal a lacuna in the treatment of Ubuntu in Jungian literature. It is common in these works to showcase a version of Ubuntu that overlooks its shadow manifestations, mirroring Tutu’s sanitized version.

Whilst Jungian scholars have used the notion of Ubuntu in creative and individualized ways, they all pay tribute to the relational aspect of Ubuntu humanism, a relationality which they see as dependent on, and affected by, Ubuntu’s commitment to forgiveness and reconciliation.

Berg (2004, 2012) sees Ubuntu as a universal way of relating that has been sadly “lost with the increasing splitting that has occurred in Western civilization” (2004, p. 246). And indeed, for Berg Ubuntu has much to teach to “the Western, so‐called ‘civilized’ world” (2004, p. 248; 2012, p. 100). Initially described as a cultural complex in 2004, Berg reconsiders her attempts to make this African notion adhere to a Western viewpoint. In 2012 she writes: “A fundamental *Weltanschauung*, like ubuntu, cannot and indeed should not be reduced in order to fit a framework that originates in another kind of cosmology” (p. 96). Included in the Interim Constitution of 1993, Berg contends that Ubuntu gave the document a particular “African flavour” (2012, p. 99), something that assisted in unifying the country. Ultimately, Berg believes that the South African reconciliation would have been impossible without Ubuntu (2004, p. 244; 2012, p. 99).

Ann Shearer refers to a well‐known passage of Tutu’s (1999) *No Future without Forgiveness*, to suggest that there exists a correspondence between the sense of interconnectedness that animates the spirit of Ubuntu and Jung’s concept of the Self, here intended as a relational field of experience. Both notions, Shearer suggests, carry the promise of the restoration of unity in a divided world. There is a “yearning for a pre‐existent ‘right order’” (2008, p. 54), Shearer contends, which emanates from the depth of the archetypal world. Restorative justice and the TRC respond to this longing by bringing people together, unlike the adversarial model of the courtroom which, writes Shearer, entraps individuals in “their persona as victim or perpetrator” (p. 51). Shearer is aware of the contentious issues surrounding the TRC: derided as the “Kleenex Commission”, the TRC was “dismissed for being too sentimental, too Christian and ineffective” (p. 54). 15 Nevertheless, Shearer contends that the “commission achieved the extraordinary when it held together a society that had been so horrendously split and created an *enduring* container for the potential meeting of opposites” (p. 54, italics mine).

Following in Shearer’s footsteps, Gottfried Heuer contraposes the conciliatory spirit of Ubuntu to the talion law as he investigates the pioneering work of psychoanalyst Otto Gross. Referring to a passage of Pamela Donleavy and Shearer’s (2008) book, Heuer writes:

Here they [Donleavy & Shearer] differentiate between the talion law predominant in Western culture with its aims of vengeance and punishments and a restorative justice characterized by efforts to rebuild a social harmony upset by unlawfulness or crime. (2010b, p. 64)

Tutu’s vision of Ubuntu as put forth in *No Future without Forgiveness* underpins Heuer’s argument against the talion law. “From a Grossian perspective,” Heuer contends, this kind of justice sharpens the “conflict between self and other, because punitive justice with its talion law is based on the will to power, whereas a restorative justice grows out of a will to relating” (2010b, p. 65).

Birgit Heuer seems to be working on a similar assumption in her exploration of the virtues of forgiveness. “In moving beyond the talion law,” Heuer writes, “*ubuntu* brings the essence of forgiveness into the socio‐political sphere by connecting it with the very essence of humanity” (2010a, p. 133; 2011, p. 58, italics in original).

The relational ethics of Ubuntu are also called upon by Thomas Singer (2016) in the context of his discussion on what Singer terms as the *Obamacare complex*. According to Singer, the Obamacare complex conceals a deeply rooted belief in social Darwinism which centres on the survival of the fittest. What would happen, muses Singer, to the polarized discussion on the Affordable Care Act if Ubuntu and not social Darwinism were the dynamic force driving the complex?

Similarly, Alan Vaughan suggests that the connective spirit of Ubuntu could function as an antidote against the systemic racism rooted in the US legal system and American cultural institutions. Ubuntu for Vaughan is kinship libido, a force that “establishes a fundamental connectivity to one another and to the Earth” (2019, p. 323; see also Vaughan, 2020).

For Roger Brooke (2008, 2019), who was raised in South Africa, Ubuntu ethics offer a significant counterbalance to Jung’s enduring suspicion towards collectives. 16 Whilst Jung viewed individuation as a solitary process that could become endangered by the demands of the collective, the ethics of Ubuntu remind us that one’s individuation is inexorably linked to that of others. Ubuntu, writes Brooke, creates the possibility for personal growth within the sustaining spirit of a community joined together by the guiding values of “inclusiveness, healing and reconciliation” as opposed to “marginalization, blame and punishment” (2019, p. 150).

In his analysis, Brooke (2008, 2019) also considers the potential constraints that a practice based on community consensus may impose on personal development. He highlights nepotism, groupthink, and a paranoid tendency to ascribe natural phenomena (such as birth defects) to the malicious intention of individuals. Whilst significant, these reflections remain few and far between and hardly developed. Brooke’s overall perspective towards Ubuntu remains one of deep reverence, as Brooke himself confirms. He contends that Ubuntu embodies “all the virtues we admire,” which include “personal responsibility, ethical self‐knowledge, strength and courage, humility, forgiveness, human understanding, a knowledge of history, and a sense of the sacred” (2008, p. 50).

This brief overview fails to fully capture the nuanced and unique arguments presented by the authors. Nevertheless, I believe it demonstrates that the current Jungian perspective has absorbed much of the sanitized version of Ubuntu popularized around the time of South Africa’s transformation. It is important for Jungians to carefully consider the ramifications of such assimilation.

Significant is the fact that the Ubuntu presented by Tutu and the TRC promoted the Christian notion of universal brotherhood precisely because there was no peace on South African soil. Ubuntu, in other words, worked on “the wrong level of aggregation” (Binsbergen, 2001, p. 450). It is as if the narrators of the new South Africa could not see “other grounds for identification between the locals […] than their belonging to humankind at large” (p. 451). 17 With the goal of eradicating deep divisions, this iteration of Ubuntu, imbued with Christian principles, aimed to downplay tensions by invoking elusive principles of common humanity, and it was in the name of these abstract ideals that the victims of apartheid were pressed to forgive.

## Troubling Forgiveness

According to Ivor Chipkin (2007), the TRC was comprized of 10 members, four of whom were ordained church ministers. Binsbergen (2001) points out that, even though the idea of Ubuntu was promoted, there were no diviner‐priests (those who uphold traditional ancestral beliefs) on the Commission. According to Binsbergen this was no accident, as the inclusion of such individuals could have presented a different perspective (potentially opposing the predominant Christian viewpoint) on concepts such as evil, sin and, more importantly, on the limits of atonement. Because, as Praeg observes: “What is a forgiveness that fails to recognize and institute against what is unforgivable?” (2014, p. 24).

The timing of forgiveness was also crucial. Berg (2014) describes Mandela as the epitome of Ubuntu (and Myers [2016, 2022] contends that he is also the embodiment of the transcendent function). Yet, Mandela’s eagerness to forgive and reconcile soon after his release from prison was perceived as an act of betrayal by the many who “needed space and time to vent their anger and express their hurt” (Praeg, 2014, p. 17). Resentment was a tool these people had at their disposal to demonstrate that they did not endorse the degrading message of the perpetrator of apartheid (Jeffrey Murphy remarks that this “is how resentment may be tied to the virtue of self‐respect” [2003, p. 77]; see also Murphy, 2005).

Scholars (Brudholm, 2006, 2008; Verdoolaege, 2006; Wilson, 2001) have noted that during the public hearings the Commission sought to discourage any display of resentment whilst at the same time encouraging “spontaneous” acts of forgiveness. When deponents attempted to convey their resentment, they were shown little empathy by the commissioners who invariably attempted to steer the conversation back to the master‐narrative of forgiveness and reconciliation. The following exchange, I believe, speaks for itself:

MR. JANTJIE: I am angry, I am not working—I have been tortured by police, I suffer, I am of ill health, I am unemployed, I suffer, my kidneys are not all right.

COMMISSIONER: We understand—we understand.

MR. JANTJIE: These people—the perpetrators they are alive, what are you doing about them—my life is ruined, what are you doing about them? They were not even jailed, I could not even go to my sister’s funeral, I was in detention. They were trying to cover up their filth—together with their Magistrate and their Judge, I was used as escape route—to cover up their filth. They wanted to save the man who had shot my sister. Who is guilty— who is guilty. Who is guilty—who is guilty. She was picked up from the streets, who is guilty—even if she had verbally offended someone—no‐one had the right to shoot her, that is nonsense. I do not want to say anymore, I’ve had it.

COMMISSIONER: Mr Nelson we understand your situation.

MR. JANTJIE: I am in pain, this police that tortured me, they are working, I am unemployed, these people walk pass me every day, the others are in De Aar—they still under employment, I cannot work for myself because of them. I don’t gain anything from that—my children they are all over the streets, they are criminals, they do not go to school.

COMMISSIONER: We understand your pain, but we ask that you try to control yourself. So that even when we ask our investigation team to find—to find out what happened, *we as the Truth Commission would like to reach a place where there can be peace and forgiveness*. 18 (October 1996, italics mine)

In some cases, victims were coerced into forgiveness, either overtly or subtly, with the belief that the act of forgiving would “liberate” them from their resentment. According to Tutu, “[f]orgiving means abandoning your right to pay back the perpetrator in his own coin, but it is a loss which liberates the victim” (1999, p. 219). But wasn’t “liberation” from the victim’s resentment precisely what the perpetrators wanted? What better outcome for the perpetrator “than to have their victims take the opiate of forgiveness and give up any hopes of justice?” (Benson, 2021, p. 864).

During the initial stages of the hearings, the commissioners often inquired about the victim’s willingness to forgive the offender, as documented by Wilson (2001) and Brudholm (2008). This request was taken seriously by many, since much was at stake in that act of forgiveness. Tutu stated that those who are unable to forgive have forfeited their Ubuntu and consequently their humanity:

We say that a human being is a human being because he belongs to a community and harmony is the essence of that community. So *ubuntu* actually *demands that you forgive*, because resentment and anger and desire for revenge undermine harmony. In our understanding, when someone doesn’t forgive, we say that person does not have *ubuntu*. That is to say, *he is not really human*. (cited in Waldmeir, 1997, p. 268, italics mine)

Just like a harsh cultural superego, Tutu’s Ubuntu demanded of the injured party to forgive or else incur punishment (the loss of humanity). In *Civilization and its Discontents*, Freud remarks that, as with the individual superego, the cultural superego makes impossible demands on people, who can barely control their natural impulses to retaliate when injured. Freud illustrates the cultural super‐ego’s “unpsychological proceedings” (1930, p.143) through his discussion of the “grandiose commandment” to love one’s neighbour and the even more perplexing commandment to love one’s enemies (p. 110). Both commandments are, according to Freud, impossible to fulfil; however, they illustrate the extent to which human aggressiveness is feared by civilization. “What a potent obstacle to civilization aggressiveness must be,” writes Freud,

if the defence against it can cause as much unhappiness as aggressiveness itself! “Natural” ethics, as it is called, has nothing to offer here except the narcissistic satisfaction of being able to think oneself better than others. (p. 143)

The extent of Freud’s scepticism is made clear in a footnote: quoting from Heinrich Heine, Freud praises the German poet’s ability to give voice “to psychological truths that are severely proscribed” (p. 110). Heine admits:

Mine is a most peaceable disposition. My wishes are: a humble cottage with a thatched roof, but a good bed, good food, the freshest milk and butter, flowers before my window, and a few fine trees before my door; and if God wants to make my happiness complete, he will grant me the joy of seeing some six or seven of my enemies hanging from those trees. Before their death I shall, moved in my heart, forgive them all the wrong they did me in their lifetime. One must, it is true, forgive one’s enemies—but not before they have been hanged. (p. 110, note 1)

Returning to Tutu’s concept of Ubuntu, the possibility of being dehumanized in the eyes of the community (due to the televised TRC hearings) likely served as a compelling motivation for forgiveness. However, it must be noted that if forgiveness is brought up prematurely, it may hinder the process of reconciliation rather than fostering it. Indeed, according to Birgit Heuer (2010a, 2011), one of the shadow aspects of forgiveness is when it is offered prematurely as a means to protect against feelings of resentment. In such cases, resentment is pushed down into the unconscious where it is left to fester, eventually posing a far greater danger. Ultimately, untimely forgiveness can potentially perpetuate resentment by reinforcing the feeling of oppression among those from whom forgiveness is being sought, and by exacerbating the power imbalance that the act of forgiveness is meant to redress (Praeg, 2014).

## Conclusion

In the Trinity essay, Jung (1948) defines the recalcitrant fourth as a challenging yet instructive psychological function that forces individuals to confront and recognize certain aspects of existence that may contradict their conscious desires and goals. The recalcitrant fourth, thus, serves as a reminder of the importance to recognize “all that lies missing outside the model of conscious social and individual integration” (Ulanov, 2007, p. 593). It was with this principle in mind that I undertook an evaluation of the use of Ubuntu in Jungian literature.

I stressed the importance of avoiding a decontextualized view of Ubuntu that fails to acknowledge its various interpretations and applications, particularly when considering the impact that this philosophical concept and practice had on the making of post‐apartheid South Africa. I noted a trend among Jungian scholars to view Ubuntu as the *exotic Other*, thereby unintentionally upholding the same colonial mindset that Jung held towards non‐European cultures.

In addition, I suggested that Jungian perspectives on Ubuntu have been greatly influenced by Tutu’s interpretation of the notion and highlighted the importance of considering the ramifications of such assimilation, especially since the archbishop utilized the concept of Ubuntu as a means of promoting national unity. Ubuntu, to clarify, was co‐opted into a political agenda with the objective of bringing together the South African population, whilst also silencing those who resented a form of reconciliation that did not tackle the systemic injustices that the South African anti‐apartheid struggle sought to correct.

The Christianized and globalized version of Ubuntu promoted by Tutu as chair of the TRC lost its local flavour; however, it still held enough symbolic resonance to strike a chord with the black community, who felt obliged to forgive and reconcile even in the absence of justice, land reform, and distribution of wealth.

Ultimately, by promoting forgiveness in the name of Ubuntu, Tutu and the TRC inadvertently turned this ancestral southern African tradition into an instrument of oppression that assisted in maintaining the status quo. Currently assimilated into Jungian literature, *this* version of Ubuntu became infused with elements of a punitive cultural superego, thereby compromising its intended purpose as an instrument for liberation.

## Footnotes

^1^
Jung’s (1948) recalcitrant fourth remains an unfamiliar concept to many in the Jungian community. Left undeveloped by Jung himself, intuited yet not properly defined, this notion appears to serve as a corrective to Jung’s own psychology which, arguably, tends to overemphasize the symbolic‐archetypal whilst downplaying the earthly‐real. As Giegerich suggests, the fourth represents Jung’s “yearning for actuality” (2020, p. 119), a desire for grounding his psychological theory in the messy reality of human relations. It is within the realm of human sociability, particularly in the tumultuous arena of politics, that this notion may be tested. Here, the tension between harmony and its disruptive forces is played out on a grand scale. Giegerich offers a critical perspective on Jung’s conception of the fourth, arguing that his “psychological *materialism*” undermines the core of psychology by prioritizing the material world over the inner one (p. 153, italics in original). Giegerich questions the relevance of the mundane, everyday world to a discipline fundamentally concerned with interiority.

^2^
According to philosopher Christoph Marx “no historical evidence has been produced to substantiate this alleged community culture” (2002, p. 52).

^3^
As with Eurocentrism, “Afrocentric approaches tend to fall into exclusive and exclusionary views” (Matolino & Kwindingwi, 2013, p. 202).

^4^
Ubuntu, write Matolino & Kwindingwi, “can be characterized as one of the narratives of return and, as such, can be linked to other narratives of return propounded by political leaders such as Kwame Nkrumah, Leopold Senghor, Julius Nyerere and Kenneth Kaunda” (2013, p. 198).

^5^
Empirical data demonstrate a correlation between the failure to pursue retributive justice and the recurrence of retributive violence (Borneman, 1997, p. 6).

^6^
George Fletcher writes that retributivism “is not to be identified with vengeance or revenge, any more than love is to be identified with lust” (cited in Walen, 2023). Crocker explains that retribution “seeks not to tame vengeance but to excise it altogether” (2002, p. 518).

^7^
Elsewhere (Cerminara, 2024), I examine the goals and methodologies of the TRC. While Jungian literature cites the TRC as a prime example of the transcendent function operating within a collective, I contend in that paper that it fails to adequately exemplify this transformative process.

^8^
Kenyan based philosopher Reginald Oduor observes that “Western thought about personhood has gone far beyond Descartes […] In fact, certain post‐Cartesian understandings of personhood, such as we find in Martin Heidegger’s ontological hermeneutics and various forms of communitarianism, particularly feminist ethics of care, are […] indistinguishable from Ubuntu” (2014, p. 219).

^9^
This prejudicial stance, writes Brooke (2019), is reflected in Jung’s theories, especially when it comes to Jung’s employment of an evolutionary model to account for the development of consciousness, a model according to which some cultures are more evolved than others.

^10^
See her memorable TED Talk of 2009. https://www.ted.com/talks/chimamanda_ngozi_adichie_the_danger_of_a_single_story?language=en

^11^
The heavily Christian emphasis of the Commission profoundly influenced the proceedings: “when victims and survivors came to our victim‐oriented hearings,” writes Tutu, “we would have a solemn atmosphere with prayers, hymns and ritual candle‐lighting […] I asked the secretariat of the world‐wide Anglican Communion to alert the nuns and monks of the religious communities of our Church to our desperate need for regular intercession” (cited in Chipkin, 2007, p. 185)

^12^
See: https://www.justice.gov.za/legislation/acts/1995-034.pdf

^13^
As philosopher Bill Wringe writes: “One does not need to regard others as morally absolved from wrongdoing […] to regard them as participants in a single political project. Anger […] is compatible with treating others as equal partners in the political community” (2022, p. 291).

^14^
Political scientist Alexander Hirsch argues that reconciliation is too loaded a term, as it conjures up the idea of a “primal unity” which in most cases never existed (2011, p. 3). Conciliation would be a more appropriate term to describe African as well as European attempts at repairing their traumatic histories.

^15^
Indeed, Innocentia Jabulisile Mhlambi refers to the TRC as a “religious theatrical display of emotional outpouring that failed victims in many ways” (2016, p. 56).

^16^
Incidentally, when it comes to discussing Jung’s Eurocentric views, some authors appear to homogenize and essentialize Europeanness, describing it as a monolithic entity. A small town of the deep south of Italy (where my paternal family originates) is more culturally connected to North Africa than, say, the industrial area of the Ruhr valley in Germany.

^17^
Implicitly this strategy acknowledged the almost “insurmountable divisions of class, somatic appearance, ethnicity, language, gender, religious denomination and political affiliation” that, contends Binsbergen, “still enter into any concrete social situation in Southern Africa” (2001, p. 451).

^18^

https://www.justice.gov.za/trc/hrvtrans/karoo/ct00513.htm
